# Optimizing phenytoin therapy: a systematic review of clinically relevant food and herb interactions

**DOI:** 10.3389/fphar.2025.1676685

**Published:** 2026-01-05

**Authors:** Adriana Monserrath Orellana-Paucar, Erick Thomas Mosquera-Lopez, Nancy Michelle Bustamante-Alvarez, María Gabriela Machado-Orellana, Daniela Alejandra Vintimilla-Rojas, John Diego Atiencia-Palacios, Ana Cristina Espinoza-Fajardo

**Affiliations:** 1 Nutrition and Dietetics School, Faculty of Medical Sciences, University of Cuenca, Cuenca, Ecuador; 2 Pharmacology and Nutritional Sciences Interdisciplinary Research Group, Faculty of Medical Sciences, University of Cuenca, Cuenca, Ecuador; 3 Medicine and Surgery School, Faculty of Medical Sciences, University of Cuenca, Cuenca, Ecuador; 4 Ministry of Public Health of Ecuador, Cuenca, Ecuador

**Keywords:** food-drug interaction, nutrient-drug interaction, herb-drug interaction, pharmacological interaction, epilepsy, phenytoin, folic acid

## Abstract

**Introduction:**

Phenytoin, a widely prescribed anticonvulsant, presents clinical challenges due to its narrow therapeutic index and potential interactions with various foods, herbs, and medications. These interactions can lead to adverse effects or subtherapeutic responses, necessitating a thorough understanding by healthcare professionals to optimize patient care.

**Methods:**

This systematic review investigates clinically significant interactions between phenytoin and dietary components. A comprehensive search across PubMed, Scopus, and the Health Virtual Library identified relevant studies published from January 1960 to December 2024. Data extraction utilized standardized forms, and evidence certainty was evaluated using the GRADE criteria.

**Results:**

Twelve of the 826 initially identified articles met the inclusion criteria. Findings revealed diverse interactions: three articles reported no interaction, five indicated enhanced drug absorption, and one noted improved drug effectiveness. Conversely, three studies documented reduced efficacy due to interactions with specific foods or herbs.

**Discussion:**

The review suggests that co-administration of phenytoin with folic acid may prevent deficiency without affecting plasma concentrations or drug efficacy. Concurrent use of phenytoin and piperine should be monitored due to potential absorption and increases in plasma levels. Additionally, it is suggested that the combination of noni and phenytoin be avoided, as it may reduce phenytoin concentrations to subtherapeutic levels. While these findings are based on studies of moderate evidence quality, further controlled clinical trials are necessary to refine pharmacological recommendations.

**Systematic Review Registration:**

CRD42018117308.

## 1 Background

The intricate interplay among medications, food, and herbal remedies presents a significant challenge in clinical practice. Food and medicinal plants, rich in diverse nutrients and bioactive compounds, have the potential to interact with pharmaceutical drugs, leading to changes in their pharmacokinetic and pharmacodynamic properties (Vuong et al., 2023). These interactions can manifest as beneficial or adverse effects, influencing the therapeutic outcome (Wang et al., 2025; Babos et al., 2021). While drug-drug interactions are often well-documented, the interactions between drugs and food or medicinal plants are typically less recognized and understood (Amadi and Mgbahurike, 2018). This knowledge gap underscores the necessity of understanding the potential for such interactions to optimize patient care and reduce the risk of adverse events.

Phenytoin, a commonly prescribed anticonvulsant, exhibits complex pharmacokinetic properties that various factors, including interactions with food and herbs, can influence. Phenytoin exerts its anticonvulsant effects primarily by stabilizing neuronal membranes and reducing neuronal excitability (Hakami, 2021). It accomplishes this by blocking voltage-gated sodium channels, which inhibits the repetitive firing of action potentials (Patocka et al., 2020) Maintaining phenytoin plasma concentrations within a narrow therapeutic range is crucial for achieving optimal seizure control while minimizing the risk of toxicity. Phenytoin is highly protein-bound, primarily to albumin. Hypoalbuminemia, a condition where albumin levels are low, can significantly increase the free fraction of phenytoin in the bloodstream. This can lead to elevated levels of pharmacologically active drugs and potentially raise the risk of toxicity, even with seemingly normal total phenytoin levels (Wilfred et al., 2022; Hong et al., 2009; Chang et al., 2020). The narrow therapeutic index of phenytoin and its susceptibility to various interactions necessitate careful monitoring and individualized dosage adjustments to ensure optimal clinical outcomes.

Phenytoin’s metabolism is crucial to its safety and efficacy profile. Understanding its metabolic pathway and potential adverse effects is essential for its safe and effective use. The CYP2C9 enzyme system primarily metabolizes phenytoin in the liver (Chang et al., 2020; Crane and Wiegand, 2023; Kan et al., 2021). Importantly, this metabolic pathway is saturable, meaning the enzyme system can be overwhelmed at higher concentrations. This saturation kinetics results in a non-linear relationship between dose and serum concentration. Even small increases in dose, when serum concentrations are already high, can cause disproportionately large increases in serum levels, significantly elevating the risk of toxicity (Wu et al., 2013).

Due to its saturable metabolism, phenytoin shows dose-dependent toxicity, with the risk of adverse effects significantly increasing as serum concentrations surpass the therapeutic range (Iorga and Horowitz, 2023). Phenytoin can lead to a wide array of adverse effects, which can be generally classified as acute or long-term. Acute adverse effects typically arise shortly after administration or with dose escalations. These may include central nervous system effects such as dizziness, ataxia, nystagmus, sedation, confusion, slurred speech, headache, and gastrointestinal disturbances like nausea and vomiting. Long-term adverse effects can develop gradually with extended use and include gingival hyperplasia, hirsutism, osteoporosis, cognitive impairment, hepatotoxicity, and severe dermatological reactions, such as Stevens-Johnson syndrome and toxic epidermal necrolysis (Patocka et al., 2020; Waterhouse and Hale, 2013; Puri et al., 2023).

Careful monitoring of adverse effects, appropriate dose adjustments, and patient education are essential to minimize the risk of complications associated with phenytoin therapy.

Despite the widespread use of phenytoin and the well-documented effects of food and herbs on drug pharmacokinetics and pharmacodynamics, there remains a lack of readily accessible, comprehensive, and evidence-based information regarding phenytoin interactions with these agents. This knowledge gap can lead to suboptimal therapeutic management, increasing the risk of adverse events and therapeutic failures in patients receiving phenytoin. To address this critical issue, the present systematic review aims to conduct a thorough and systematic analysis of the existing literature on phenytoin interactions with food and herbs. The goal is to provide healthcare professionals with a comprehensive and evidence-based resource to guide clinical decision-making, promote preventive measures, and ultimately optimize therapeutic outcomes for patients prescribed phenytoin.

## 2 Methods

### 2.1 Guidelines and protocol

This systematic review followed the Preferred Reporting Items for Systematic Reviews statement (PRISMA) (Page et al., 2021). It adhered to the protocol titled “Interactions of clinical relevance associated with the concurrent administration of prescription drugs and food or medicinal plants: a systematic review protocol” (PROSPERO registration number CRD42018117308) (Orellana et al., 2020).

### 2.2 Search strategy

The search strategy was developed using the Patient/Problem, Intervention, Comparison group, and Outcome (PICO) framework, focusing on patients with epilepsy or individuals receiving phenytoin therapy (Population), examining phenytoin co-administration with food or herbal products (Intervention/Exposure), compared with phenytoin administration without such co-exposures (Comparator), to determine clinical outcomes related to pharmacokinetic and pharmacodynamic interactions (Outcomes), using human studies including clinical trials, observational studies, and case reports (Study designs). A comprehensive literature search was conducted from January 1960 to December 2024 across three databases: PubMed, Scopus, and the Virtual Health Library (VHL). The search employed a combination of Medical Subject Headings (MeSH) and keywords, such as “food-drug interaction,” “plant-drug interaction,” “herbal-drug interaction,” “phenytoin,” and “epilepsy.” Duplicate articles were identified and removed. Additionally, a lateral search of the reference lists from selected studies in PubMed was performed to minimize the potential for omitting relevant articles.

### 2.3 Selection and exclusion criteria

Three independent reviewers conducted the article selection process for this systematic review. Studies were deemed eligible for inclusion if they met the following criteria: (a) published in English or Spanish; (b) original, full-text articles; (c) conducted in human subjects across all age groups (from birth to ≥65 years); (d) explored the pharmacological interactions between phenytoin and concurrently administered food or herbal products; and (e) used a case report, clinical trial, comparative, or observational study design. Articles were excluded if they: (a) were published in a language other than English or Spanish; (b) were review articles; (c) involved cell culture, tissue-based, or animal research; or (d) were duplicates.

### 2.4 Data extraction

Data extraction from the selected studies was performed independently by three reviewers using a standardized spreadsheet. Any discrepancies in data entry were resolved through consensus following a re-review of the original article. The following data elements were extracted:

- Publication details: authors, year of publication, language, title, journal, country where the study was conducted, and funding sources;
- Study design: type of study (e.g., clinical trial, quasi-experimental, case report, case-control, cohort, observational), methods of participant recruitment, and data collection methods;
- Study objectives, outcomes, and outcome measures;
- Participant characteristics: sample size, demographic and socioeconomic characteristics, age group stratification (children <18 years, adults 19–65 years, older adults >65 years), and specific physiological conditions (e.g., pregnancy, lactation);
- Phenytoin: daily dose administered;
- Pharmacological interaction: type of interaction (food-drug or herb-drug);
- Food: type of food (e.g., fruits, vegetables, dairy), specific food item (e.g., cow’s milk, beef), scientific name (for fruits and vegetables), and reported amount consumed;
- Herbs: scientific name of the plant, type of herbal preparation (e.g., infusion, herbal product), and reported amount consumed or dose of active constituents administered;
- Safety issues: reported adverse reactions; and
- Study limitations: potential biases (e.g., response bias, selection bias) and other limitations.

### 2.5 Evaluation of the certainty of evidence

The Grading of Recommendations, Assessment, Development, and Evaluation (GRADE) system was used to thoroughly assess the certainty of evidence across the studies included in this review (Schünemann et al., 2013). The GRADE framework emphasizes five domains: risk of bias, inconsistency, indirectness, imprecision, and publication bias. Based on these domains, the quality of evidence was classified as high, moderate, low, or very low.

### 2.6 Data synthesis

Data synthesis was performed using a narrative approach, providing a comprehensive summary of the findings from the selected studies. This narrative synthesis emphasized a critical analysis of the reliability of the evidence from the twelve chosen articles to clarify the clinical characteristics and safety concerns related to pharmacological interactions between phenytoin and food or herbal products.

## 3 Results

### 3.1 Search results

**FIGURE 1:**
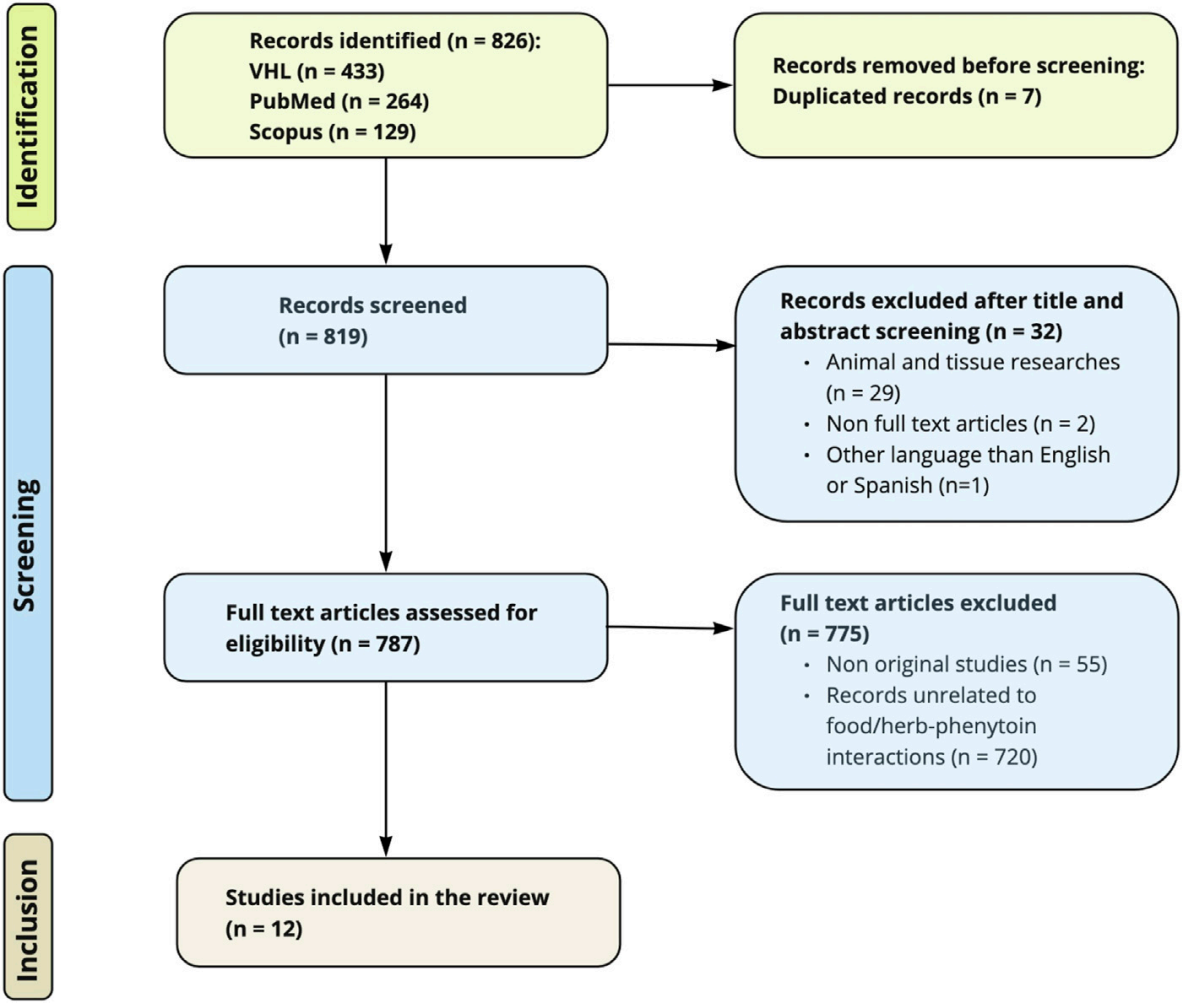
Flow chart based on the PRISMA statement.

The initial database searches yielded 826 articles (VHL: 433, PubMed: 264, Scopus: 129). After removing seven duplicates, 819 articles underwent title and abstract screening. Of these, 32 records were excluded for not meeting the eligibility criteria. A full-text review was conducted on the remaining 787 articles, excluding 775 articles that were either not original research articles or did not focus on interactions between phenytoin and food or herbs. Twelve studies fulfilled the predefined selection criteria and were included in this systematic review. The selected studies comprised four randomized controlled trials, four quasi-experimental studies, and four case reports. Figure 1 provides a visual representation of the study selection process.

### 3.2 Identified interactions

Of the twelve included studies, three reported no pharmacological interactions between phenytoin and the investigated nutrients/foods or herbs (Hernández et al., 2005; Doak et al., 1998; Cook et al., 2001). Five studies described nutrient/food-phenytoin interactions that enhanced drug absorption (Melander et al., 1979; Velpandian et al., 2001; Berg et al., 1995; Bano et al., 1987; Pattanaik et al., 2006), while one reported improved drug effectiveness (Martikainen et al., 2012). Conversely, three studies documented decreased phenytoin’s anticonvulsant effect due to food/herb-phenytoin interactions (Kang et al., 2015; Longe and Smith, 1988; Kupiec and Raj, 2005). Tables 1–4 provide detailed information regarding these studies’ findings.

**TABLE 1:** Summary of selected articles that report no interaction between phenytoin and nutrients or food.

Article reference	Type of study	Aim(s)	Population	Results	Prescribed drugs	Pharmacological interaction and dosage	Study limitations
Hernández et al. (2005)	Randomized Controlled Trial	To describe the effect of folic acid depletion on cognition in patients with epilepsy treated with phenytoin and carbamazepine	Eighteen patients aged 18–50 years with a diagnosis of cryptogenic epilepsy	No changes were observed in serum antiepileptic drug concentration or seizure frequency following folic acid administration	Experimental group: phenytoin 340 mg/day (mean dose) Placebo group: phenytoin 360 mg/day (mean dose)	Food-Drug Interaction Folic acid 5 mg/day for 6 months	The study did not involve dose-response gradient and did not control for potential confounding factors, including diet, herbal tea consumption, and the use of self-prescribed medications
Doak et al. (1998)	Randomized Controlled Trial	To compare the bioavailability of phenytoin sodium solution and phenytoin acid suspension in healthy volunteers receiving continuously infused enteral feeding	Ten healthy volunteers aged 23–43 years	No significant difference in bioavailability is observed between phenytoin sodium solution and phenytoin acid suspension when administered with continuous enteral feeding	Phase A: Phenytoin sodium solution (435 mg) i.v.; Phase B: Phenytoin acid suspension (400 mg) through a nasogastric tube; Phase C: Phenytoin sodium solution (435 mg) via nasogastric tube	Food-drug Interaction Isocal: 240 mL formula as a bolus followed by a continuous infusion (100 mL/h) by nasogastric tube	The study design limits generalizability due to differences in phenytoin administration route compared to long-term treatment in outpatients, single-dose and nonblinded assessment, and recruitment of healthy volunteers. Furthermore, the sample size is minor (n = 10). Adherence to treatment varies: two participants drop out the study
Cook et al. (2001)	Randomized Controlled Trial	To investigate the possible influence of a high-fat meal on the bioavailability and pharmacokinetics of phenytoin (Dilantin Kapseals) in healthy volunteers	Twenty-four healthy volunteers (10 men and 14 women); aged 29–69 years	No significant differences in maximum concentration or extent of absorption were observed between fed and fasted states	Fed and fasted conditions: 100 mg phenytoin (Dilantin Kapseal)	Food-Drug Interaction A breakfast consisting of 150 kcal protein, 250 kcal carbohydrate, and 500–600 kcal fat was given	Study generalizability is limited by the use of healthy volunteers and a single-dose design, which may not reflect phenytoin bioavailability in patients with epilepsy receiving long-term treatment

**TABLE 2:** Summary of selected articles on enhanced drug absorption because of nutrient/food-phenytoin pharmacokinetic interactions.

Article reference	Type of study	Aim(s)	Population	Results	Prescribed drugs	Pharmacological interaction and dosage	Study limitations
Melander et al. (1979)	Quasi-Experimental Study	To determine the possible influence of mealtime on the bioavailability of phenytoin	Eight healthy volunteers (1 woman and 7 men); aged 23–27 years	Phenytoin absorption is accelerated when administered with a 440 kcal meal (20% protein, 35% fat, 45% carbohydrates)	Phenytoin 300 mg	Food-Drug Interaction Fast conditions: 50 mL of water Fed conditions: 150 mL of low-fat milk, 100 mL of orange juice, one egg, two slices of bread, 5 g of margarine, 20 g of orange marmalade, 20 g of cheese, and 100 mL of unsweetened coffee	The study was conducted on a minor sample (n = 8) of healthy volunteers, and the process of selecting participants was not specified A single drug dose may not adequately predict the bioavailability of phenytoin
Velpandian et al. (2001)	Quasi-Experimental Study	To analyze the effect of the consumption of food containing piperine (*Piper nigrum)* on the pharmacokinetics of phenytoin	Six healthy volunteers (2 women and 4 men). No information regarding age was provided	Piperine from pepper may increase phenytoin absorption and delay its elimination, potentially by inhibiting drug metabolism	Phenytoin 300 mg, 30 min after breakfast	Food-Drug Interaction. Experimental group: 200 mL of soup containing tomato, tamarind, coriander, legumes, garlic, fennel, asafoetida, and pepper (1 g/200 mL soup) Control group: 200 mL of soup (same composition except for pepper)	The process for selecting study participants was not specified. The evaluation focused on a minor sample (n = 6) of healthy volunteers, which limits the generalizability of the findings. Results were not assessed based on a dose-response relationship
Berg et al. (1995)	Randomized Controlled Trial	To examine the interdependence of phenytoin and folic acid in six healthy women	Six healthy female volunteers; aged 28–39 years	In comparing two treatment approaches, treatment 2, which was associated with increased serum folic acid levels, resulted in faster attainment of phenytoin steady state compared to treatment 1, which was associated with decreased serum folic acid levels	Treatment 1: 300 mg sodium phenytoin/day for 22 days Treatment 2: 300 mg sodium phenytoin plus folic acid daily for 22 days	Food-Drug Interaction Treatment 1: No dose of folic acid was administered Treatment 2: An oral dose of 1 mg/day of folic acid was administered in addition to phenytoin	Limitations of this study include the recruitment of only healthy volunteers, an imprecise selection method for study subjects, and deficient control over the participants’ daily diets
Bano et al. (1987)	Quasi- Experimental Study	To investigate the influence of piperine on phenytoin kinetics in healthy volunteers	Five healthy male volunteers; aged 25–40 years	Co-administration of piperine enhanced phenytoin absorption	Phenytoin 300 mg	Food-Drug Interaction. Piperine: 20 mg	The study employed a single-dose design conducted exclusively on healthy volunteers. The selection method for participants was imprecise and the small sample size limits the generalizability of the findings. There was a lack of control for potential confounding factors, such as diet, herbal tea consumption, and the use of self-prescribed medications
Pattanaik et al. (2006)	Quasi- Experimental Study	To explore the effect of a single dose of piperine in patients with uncontrolled epilepsy on the steady-state pharmacokinetics of phenytoin	Twenty patients aged 20–45 years with a diagnosis of epilepsy	Piperine increased significantly the mean plasma concentration of phenytoin at most of the time points in both dose groups assessed	Phenytoin 150 mg or 200 mg twice daily	Food-Drug Interaction. Piperine: 20 mg	The selection process of participants was not described. There was insufficient control over potential confounding factors, including diet, consumption of herbal teas, and the use of self-prescribed medications

**TABLE 3:** Overview of the chosen article on enhanced drug effectiveness resulting from the pharmacodynamic interaction between phenytoin and food.

Article reference	Type of study	Aim(s)	Population	Results	Prescribed drugs	Pharmacological interaction and dosage	Study limitations
Martikainen et al. (2012) (Martikainen et al., 2012)	Case Report	To describe the benefits of combining phenytoin, oxcarbazepine, and levetiracetam with a low glycemic index diet in treating epilepsy related to mutations in the mitochondrial polymerase gamma gene (POLG)	One woman with severe headaches, visual flashing, speech difficulty and generalised seizures; aged 26 years	A combination therapy of phenytoin, oxcarbazepine, levetiracetam, and a low-glycemic-index diet was both effective and well-tolerated in a patient with severe episodes of POLG-related mitochondrial epilepsy with nonconvulsive status epilepticus	No dose of phenytoin was reported	Food-Drug Interaction Dietary treatment with a low percentage of carbohydrates. Carbohydrate-containing foods have a low glycemic index (GI: <50) relative to glucose	Dosing information for phenytoin, oxcarbazepine, and levetiracetam was not provided. The specific composition of the low glycemic index diet was not detailed

**TABLE 4:** Summary of selected articles reporting the reduction of drug effects caused by pharmacodynamic interactions between phenytoin and herbs or food.

Article reference	Type of study	Aim(s)	Population	Results	Prescribed drugs	Pharmacological interaction and dosage	Study limitations
Kang et al. (2015)	Case Report	To report an unsafe herb-drug interaction between noni juice and phenytoin	One man with a diagnosis of epilepsy; aged 49 years	Consumption of noni juice was associated with subtherapeutic phenytoin levels and subsequent loss of seizure control	Without noni juice: 600 mg/day of phenytoin was administered during the first 7 days. On day 11, the dose was readjusted to 500 mg/day With noni juice: during the first 14 days, 500 mg/day of phenytoin was administered	Herb-Drug Interaction The patient reported consuming 80–100 mL of noni juice twice daily for 10 years	Limited generalizability due to single-patient approach
Longe and Smith (1988)	Case Report	To describe the case of an elderly patient hospitalized for pneumonia who experienced the loss of seizure control due to an interaction between an oral supplement (Ensure) and phenytoin	One man with a diagnosis of chronic obstructive pulmonary disease and multiple episodes of pneumonia; aged 62 years	Oral feeding with Ensure was associated with subtherapeutic plasma levels of phenytoin, leading to loss of seizure control	Pre-hospitalization: 300 mg of phenytoin and 120 mg of phenobarbital. Hospitalization 260 mg (average value) and 120 mg of phenobarbital Post-hospitalization: 325 mg phenytoin (average value) and 30 mg phenobarbital	Food-Drug Interaction. Three daily doses of 240 mL of Ensure were administered for 7 days	Given that this was an elderly patient with several comorbidities, the findings may not be relevant to younger patients diagnosed with epilepsy. Additionally, there was no information provided about potential confounding factors, such as the nutritional alternative prescribed in place of Ensure
Kupiec and Raj (2005)	Case Report	To inform the case of an elderly patient who suffered a faltal breakrhrough seizure due to herb-drug interaction among the prescribed anticonvulsants (phenytoin and valproic acid) and *Ginkgo biloba*	One man with a diagnosis of epilepsy; aged 55 years	Autopsy results revealed that the serum levels of both anticonvulsants were below therapeutic ranges	No doses of anticonvulsant drugs were specified	Herb-Drug Interaction. No dose of *Ginkgo biloba* was informed	Single-patient approach. Unspecified dietary information. The source of information regarding the administration of non-prescribed drugs (including *Ginkgo biloba*) was not specified. The administered doses of phenytoin and *Ginkgo biloba* were not mentioned

### 3.3 Certainty of evidence evaluation

One clinical trial was rated as having moderate-quality evidence (Hernández et al., 2005), while the other three randomized clinical trials (RCTs) were assessed as having very low-quality evidence (Doak et al., 1998; Cook et al., 2001; Berg et al., 1995). Similarly, three quasi-experimental studies were classified as very low-quality evidence (Melander et al., 1979; Velpandian et al., 2001; Bano et al., 1987), and only one was rated as moderate quality (Pattanaik et al., 2006). Among the case reports, one was assessed as moderate quality (Kang et al., 2015), and three were considered very low quality (Martikainen et al., 2012; Longe and Smith, 1988; Kupiec and Raj, 2005). Table 5 provides a detailed rationale for the GRADE rating of each included study based on nine components: study design, risk of bias, inconsistency, indirectness, imprecision, publication bias, magnitude of effect, dose-response gradient, and confounding factors.

**TABLE 5:** Summary of GRADE evaluation criteria for the selected articles.

Reference	1. Study design	2. Risk of bias	3. Inconsistency	4. Indirectness	5. Imprecision	6. Publication bias	7. Magnitude of effect	8. Dose-response gradient	9. Confounding factors	GRADE final rating
Hernández et al. (2005)	Strong	Moderate	Strong	Strong	Strong	Strong	Moderate	Weak	Weak	Moderate
Doak et al. (1998)	Strong	Weak	Weak	Weak	Weak	Strong	Weak	Weak	Moderate	Very Low
Cook et al. (2001)	Strong	Weak	Strong	Weak	Weak	Strong	Weak	Weak	Strong	Very Low
Melander et al. (1979)	Moderate	Weak	Weak	Weak	Weak	Strong	Weak	Weak	Strong	Very Low
Velpandian et al. (2001)	Moderate	Weak	Strong	Weak	Weak	Strong	Weak	Weak	Strong	Very Low
Berg et al. (1995)	Strong	Weak	Strong	Weak	Weak	Strong	Weak	Weak	Strong	Very Low
Bano et al. (1987)	Moderate	Weak	Strong	Weak	Weak	Strong	Weak	Weak	Weak	Very Low
Pattanaik et al. (2006)	Moderate	Weak	Strong	Strong	Strong	Strong	Moderate	Strong	Weak	Moderate
Martikainen et al. (2012)	Weak	Weak	Strong	Strong	Weak	Strong	Weak	Weak	Moderate	Very Low
Kang et al. (2015)	Weak	Moderate	Strong	Strong	Weak	Strong	Moderate	Strong	Strong	Moderate
Longe and Smith (1988)	Weak	Weak	Strong	Strong	Weak	Strong	Weak	Strong	Weak	Very Low
Kupiec and Raj (2005)	Weak	Weak	Strong	Strong	Weak	Strong	Weak	Weak	Weak	Very Low

## 4 Discussion

Phenytoin, a widely prescribed antiepileptic drug since 1938, remains a first-line treatment option for epilepsy due to its established efficacy. Its effectiveness derives from its ability to block both voltage-gated and frequency-gated sodium channels for longer durations than other antiepileptic drugs (AEDs), effectively suppressing high-frequency neuronal firing. Additionally, phenytoin is thought to increase brain levels of inhibitory neurotransmitters such as serotonin and gamma-aminobutyric acid, promoting functional equilibrium at the neuronal membrane level (Besag and Vasey, 2021).

Despite its therapeutic benefits, phenytoin’s narrow therapeutic index and potential adverse effects, especially with long-term use, require careful consideration. Cognitive impairment affects about 70% of individuals with epilepsy, and phenytoin has been linked to worsening this issue (Novak et al., 2022). In addition to its direct neurocognitive effects, phenytoin has been shown to lower serum folate concentrations, which may contribute to cognitive decline. Phenytoin affects folate absorption and metabolism, decreasing plasma folate levels (Kathiravan et al., 2021). Importantly, folate administration does not seem to influence phenytoin plasma concentrations, as highlighted in a moderate-quality RCT (Hernández et al., 2005) Including patients with epilepsy in the study enhances the clinical relevance of these findings. However, confounding factors like herb or food consumption were not controlled, and there was no assessment of a dose-response gradient. Conversely, a very low-quality RCT examining the co-administration of phenytoin and folic acid found that folate supplementation helped achieve steady-state phenytoin levels faster, indicating improved AED absorption (Berg et al., 1995). The study’s drawbacks included recruiting only healthy volunteers, an imprecise participant selection method, and insufficient control over daily diets. Considering both pieces of evidence, particularly the moderate-quality one, folate administration seems beneficial for patients on chronic phenytoin therapy to reduce the risk of folate deficiency without impacting AED plasma concentrations.

A case report documented a reduction in phenytoin plasma concentration to subtherapeutic levels, leading to a loss of seizure control attributed to the consumption of noni juice (*Morinda citrifolia*) (Kang et al., 2015). Although it is a single case study, it presents moderate evidence certainty due to the observed dose-response relationship supporting this interaction.

Unlike the previous case with noni, the low-rated certainty of the evidence in the case report regarding a possible interaction between phenytoin and *Ginkgo biloba* is mainly based on the single-patient approach, the absence of dietary information, and the fact that the source of information regarding the administration of *Ginkgo biloba* was not specified (Kupiec and Raj, 2005). Furthermore, the dosages of phenytoin and *Ginkgo biloba* given to the patient were not mentioned. Thus, solid data is insufficient to discourage this co-administration.

The findings on the co-administration of phenytoin with food show inconsistencies among the selected studies. A very low-quality RCT reported no significant influence of continuous enteral feeding on phenytoin plasma concentrations (Doak et al., 1998). This observation aligns with the results of another low-rated RCT, suggesting that a high-fat meal does not impact the pharmacokinetics or bioavailability of phenytoin (Cook et al., 2001). However, both studies have significant limitations, primarily due to the inclusion of a small sample of healthy volunteers and a single-dose approach. These findings contrast with the accelerated drug absorption noted in a very low-quality quasi-experimental study when phenytoin was administered with a meal comprising 20% protein, 35% fat, and 45% carbohydrates (Melander et al., 1979). However, this study also faces critical limitations, including sample size and focus on healthy subjects. The participant selection process was not detailed, and the single-dose method is inadequate for predicting phenytoin’s bioavailability. Further discrepancies arise when considering case reports with lower evidence (Martikainen et al., 2012; Longe and Smith, 1988). One study indicated that a low-glycemic index diet enhanced the effectiveness of phenytoin in managing POLG-related mitochondrial epilepsy, demonstrating improved control of status epilepticus (Martikainen et al., 2012). Conversely, another article reported an interaction between Ensure and phenytoin, which reduced AED plasma concentration to subtherapeutic levels (Longe and Smith, 1988). In summary, the available scientific evidence regarding potential interactions between phenytoin and food remains inconclusive, lacking the robustness to recommend or discourage co-administration.

Furthermore, two very low-quality quasi-experimental studies suggested that piperine may enhance phenytoin absorption and delay its elimination (Velpandian et al., 2001; Bano et al., 1987). The main limitations of both studies include a single-dose design with a small sample size of healthy volunteers and a lack of control over confounding factors. A third study, considered moderate quality, supports piperine’s role in improving phenytoin absorption (Pattanaik et al., 2006). This potential interaction is backed by evidence that piperine has similar effects on the absorption of other compounds, including curcumin (Pawar et al., 2021). Therefore, co-administering phenytoin and piperine appears beneficial for enhancing drug absorption, based on the moderate quality of evidence from this third study, primarily supported by the dose-response evaluation.

## 5 Conclusions and future directions

While drug-drug interactions associated with phenytoin are well-established, interactions between this anticonvulsant and herbs or food are less known. Given phenytoin’s narrow therapeutic index (10–20 mg/mL), it is crucial to disseminate scientific information to prevent any interactions that could alter its plasma concentration.

Given the moderate scientific quality from the available RCTs, co-administration of folic acid with phenytoin may be beneficial in preventing folate deficiency in patients receiving long-term treatment (Hernández et al., 2005). Although phenytoin has been associated with reduced folate levels, current evidence indicates that folic acid supplementarion does not significantly alter phenytoin pharmacokinetics. Accordingly, correction of folate deficiency may help mitigate potential cognitive decline in individuals with epilepsy treated with phenytoin; however, this relationship remains insufficiently demonstrated and should be interpreted with caution due to study limitations, including the lack of dose-response evaluation and inadequate control for dietary intake, herbal tea consumption, and self-prescribed medications. Future clinical studies examining phenytoin-folate interactions should employ rigorous methodological designs, including control of key confounding factors and standardized outcome measures, to strengthen the evidence base and better inform clinical recommendations.

Given the moderate level of scientific evidence presented in the case report, it is advisable to avoid co-administering noni and phenytoin whenever possible (Kang et al., 2015). As this clinical observation is only backed by preclinical studies (Chandra and Veeresham, 2011) conducting randomized, double-blind clinical trials is crucial to ascertain the effect of noni on phenytoin pharmacokinetics with a higher degree of scientific certainty.

Currently, there is no scientifically robust evidence to support or discourage the co-administration of phenytoin with food. Rigorous RCTs are needed to effectively control key variables and better understand this type of interaction. For instance, future studies should be designed to identify specific macro- or micronutrients responsible for any observed interactions.

The studies analyzed in this review regarding the potential of piperine to enhance the absorption and bioavailability of phenytoin are contradictory (Velpandian et al., 2001; Bano et al., 1987; Pattanaik et al., 2006). Based on the moderate-quality research (Pattanaik et al., 2006) this review recommends monitoring the plasma concentrations of the anticonvulsant when co-administered with piperine due to the potential inhibition of microsomal enzymatic metabolism of phenytoin triggered by piperine (Gohil and Mehta, 2009). Therefore, despite the proposed interaction, additional clinical studies are needed to determine the clinical significance of this potential interaction.

This review highlights the intricate nature of phenytoin interactions and emphasizes the need for further research to establish clear clinical guidelines. While supplementing with folic acid seems to be a reasonable strategy to counter phenytoin-induced folate deficiency, it is also advisable to avoid using noni alongside phenytoin. Furthermore, the combination of phenytoin and piperine appears to increase drug plasma concentration, which must be monitored closely due to phenytoin’s narrow therapeutic index. Future research should utilize rigorous methodologies, including randomized controlled trials with adequate sample sizes, standardized protocols, and comprehensive efficacy and safety outcomes assessments. By addressing these knowledge gaps, healthcare providers can improve phenytoin treatment and positively affect the lives of those living with epilepsy.

## Data Availability

The original contributions presented in the study are included in the article/supplementary material, further inquiries can be directed to the corresponding author.
